# Extending the study of visual attention to a multisensory world (Charles W. Eriksen Special Issue)

**DOI:** 10.3758/s13414-020-02061-8

**Published:** 2020-05-17

**Authors:** Charles Spence

**Affiliations:** 1 Crossmodal Research Laboratory, University of Oxford, Oxford, UK; 2 Department of Experimental Psychology, Anna Watts Building, University of Oxford, Oxford, OX2 6GG UK

**Keywords:** Eriksen, Flanker task, Zoom lens, Visual, Spatial attention, Crossmodal

## Abstract

Charles W. Eriksen (1923–2018), long-time editor of *Perception & Psychophysics* (1971–1993) – the precursor to the present journal – undoubtedly made a profound contribution to the study of selective attention in the visual modality. Working primarily with neurologically normal adults, his early research provided both theoretical accounts for behavioral phenomena as well as robust experimental tasks, including the well-known Eriksen flanker task. The latter paradigm has been used and adapted by many researchers over the subsequent decades. While Eriksen’s research interests were primarily focused on situations of unimodal visual spatially selective attention, here I review evidence from those studies that have attempted to extend Eriksen’s general approach to non-visual (i.e., auditory and tactile) selection and the more realistic situations of multisensory spatial attentional selection.

## Introduction: Visual spatially selective attention

As an undergraduate student studying Experimental Psychology at Oxford University at the end of the 1980s, I was taught, or rather tutored, by the likes of Alan Allport (e.g., Allport, 1992; Neumann, Van der Heijden, & Allport, 1986), Peter McLeod (e.g., McLeod, Driver, & Crisp, 1988), and the late Jon Driver (e.g., Driver, 2001; Driver, McLeod, & Dienes, 1992; Driver & Tipper, 1989). At the time, the study of visual attention was a core component of the *Human Information Processing* (HIP) course. The research of Steve Tipper (e.g., Tipper, 1985; Tipper, Driver, & Weaver, 1991; Tipper, Weaver, Jerreat, & Burak, 1994) and Gordon Baylis (e.g., Baylis & Driver, 1993), then both also based in the Oxford department, helped to keep the focus of attention research squarely on the visual modality. The spotlight of attention (note the distinctly visual metaphor; Eriksen & Hoffman, 1973), and the Eriksen flanker task (B. A. Eriksen & C. W. Eriksen, 1974), were often mentioned. Indeed, many an undergraduate essay discussed the modifications to the conception of visual selective attention that had been facilitated by developments added to the cognitive psychology paradigms introduced by Charles W. Eriksen and his collaborators in the 1970s and 1980s (see LaBerge, 1995; and Styles, 2006, for a review).

This change in focus from early *auditory* selective attention research (e.g., on, or at least inspired by, the cocktail party situation; Cherry, 1953; Conway, Cowan, & Bunting, 2001; Moray, 1959, 1969b; see Bronkhorst, 2000, for a review) was brought about, at least in part, by the arrival of the personal computer (see Styles, 2006). While similarities and differences between the mechanisms of selective attention operating in the auditory and visual modalities were occasionally commented on in the review papers that we were invited to read as undergraduates (e.g., see Moray, 1969a),^1^ the view of attention was seemingly only ever a unimodal, or unisensory, one. I have devoted my own research career to the question of attentional selection in a *multisensory* world (e.g., see Spence, 2013; Spence & Driver, 2004; Spence & Ho, 2015a, b).^2^ The question that I would like to address in this review, therefore, is how well Eriksen’s paradigms, not to mention the insights and theoretical accounts that were based on them subsequently, stand-up in a world in which spatial attentional selection is, in fact, very often multisensory (e.g., see Soto-Faraco, Kvasova, Biau, Ikumi, Ruzzoli, Morís-Fernández, & Torralba, 2019; Theeuwes, van der Burg, Olivers, & Bronkhorst, 2007).

### The Eriksen flanker task

The Eriksen flanker task (Eriksen & Eriksen, 1974) was a popular paradigm amongst cognitive psychologists in Oxford and elsewhere. Indeed, as of the end of 2019, the paper had been cited more than 6,000 times. The original study involved participants making speeded discrimination responses to a visual target letter that was always presented from just above fixation, while trying to ignore any visual flanker stimuli that were sometimes presented to either side of the target. Of course, under such conditions, one can easily imagine how both overt and covert spatial attention would have been focused on the same external location (see Spence, 2014a, for a review). Indeed, the fixing of the target location was specifically designed to eliminate the search element that likely slowed participants’ responses in the other visual noise studies that were published at around the same time (e.g., Colegate, Hoffman, & Eriksen, 1973; Eriksen & Collins, 1969; Eriksen & Schultz, 1979; Estes, 1972). Treisman’s visual search paradigm, by contrast, focused specifically on the “search” element. That said, the distractors in the latter’s studies would very often share features with the target (see Treisman & Gelade, 1980; Treisman & Souther, 1985). The 1-s monocular presentation of the visual target and distractor letters in Eriksen and Eriksen’s (1974) study was achieved by means of a tachistoscope (at this point, the widespread introduction of the personal computer to the field of experimental psychology was still a few years off). The six participants (this being a number that would be unlikely to cut the mustard with assiduous reviewers these days) who took part in the study were instructed to pull a response lever in one direction for the target letters “H” and “K,” and to move the lever in the opposite direction if the target letters “S” and “C” should be presented at the target location instead. Eriksen and Eriksen (1974) varied whether or not there were any visual distractors, and if so, how they were related to the target (see Table 1 for a summary of the conditions tested in their study). Of particular interest, the distractor (or noise) letters could be congruent or incongruent with the target letter, or else unrelated (that is, not specifically associated with a response). The spatial separation between the seven letters in the visual display was varied on a trial-by-trial basis.

**Table 1:** Experimental conditions and representative displays. [Reprinted from Eriksen & Eriksen (1974)]

Condition	Example						
1 Noise same as target	H	H	H	*H*	H	H	H
2 Noise response compatible	K	K	K	*H*	K	K	K
3 Noise response incompatible	S	S	S	*H*	S	S	S
4 Noise heterogeneous—Similar	N	W	Z	*H*	N	W	Z
5 Noise heterogeneous—Dissimilar	G	J	Q	*H*	G	J	Q
6 Target alone				H			

The results revealed that speeded target discrimination reaction times (RTs) decreased as the target-distractor separation increased (from 0.06°, 0.5°, to 1.0° of visual angle). As the authors put it: *“In all noise conditions, reaction time (RT) decreased as between-letter spacing increased.”* In fact, the interference effects were greatest at the smallest separation, with performance at the other two separations being more or less equivalent. Eriksen and Eriksen went on to say that: *“However, noise letters of the opposite response set were found to impair RT significantly more than same response set noise, while mixed noise letters belonging to neither set but having set-related features produced intermediate impairment”* (Eriksen & Eriksen, 1974, p. 143).

When thinking about how to explain their results, Eriksen and Eriksen (1974, p. 147) clearly stated that the slowing of participants’ performance was *“not a sort of ‘distraction effect’.”* Nowadays, I suppose, one might consider whether their effects might, at least in part, be explained in terms of crowding instead (e.g., Cavanagh, 2001; Tyler & Likova, 2007; Vatakis & Spence, 2006), given the small stimulus separations involved. At the time their study was published, the Eriksens, wife and husband, argued that their results were most compatible with a response compatibility explanation (see also Miller, 1991).

Subsequently, it has been noted that the participants in Eriksen and Eriksen’s (1974) original study could potentially have resolved the task that they had been given on the basis of simple feature discrimination (i.e., curved vs. angular lines), rather than by necessarily having to discriminate the letters themselves (see Watt, 1988, pp. 127-129).^3^ Addressing this potential criticism, C. W. Eriksen and B. A. Eriksen (1979) had their participants respond to the target letters “H” and “S” with one response key and to the letters “K” and “C” with the other. The results of increasing the target-distractor (or -noise) distance were, however, the same as in their original flanker study.

Another potential concern with the original Eriksen flanker task relates to the distinction between the effects of overt and covert visual attentional orienting (e.g., Remington, 1980; Shepherd, Findlay, & Hockey, 1986). Indeed, Hagenaar and van der Heijden (1986) suggested that the distance effects reported in Eriksen and Eriksen’s (1974) seminal study might actually have reflected little more than the consequences of differences in visual acuity. That is, they suggested that distant distractors might have interfered less simply because they were presented in regions of the visual field with lower acuity (see also Jonides, 1981a). That said, subsequent research by Yantis and Johnston (1990), Driver and Baylis (1991), and many others showed that acuity effects did not constitute the whole story as far as flanker interference is concerned. The latter researchers presented the target and distractor letters on a virtual circle centred on fixation (to equate acuity). By pre-cuing the likely target location, they were able to demonstrate an effect of target-distractor separation despite the fact that visual acuity was now equivalent for all stimuli (i.e., regardless of the distance between the target and distractors). Interestingly though, Driver and Baylis argued that their results did not fit easily with Treisman’s Feature Integration Theory (FIT; see Treisman & Gelade, 1980).^4^

In summary, while it is undoubtedly appropriate for researchers to try and eliminate the putative effects of changes in visual acuity on performance, and to try and ensure that the participants really are discriminating between letters rather than merely line features, the basic flanker effect has remained surprisingly robust to a wide range of experimental modifications (improvements) to Eriksen and Eriksen’s original design (see also Miller, 1988, 1991).

Elsewhere, my former supervisor, Jon Driver, used a modified version of the Eriksen flanker task in order to investigate questions of proximity versus grouping by common fate in the case of visual selective attention (Driver & Baylis, 1989). In this series of four studies, a row of five letters was presented, centered on fixation. Once again, the participant’s task involved trying to discriminate the identity of the central target letter, and ignore the pair of letters presented on either side. In this case, though, the target letter sometimes moved downward with the outer distractors, while the inner distractors remained stationary. The results revealed that the Gestalt grouping by common motion (e.g., Kubovy & Pomerantz, 1981; Spence, 2015; Wagemans, 2015) determined flanker interference rather than the absolute distance between the target and the distractor, as might have been suggested by a simple reading of the attentional spotlight metaphor. In a related vein, some years earlier, Harms and Bundesen (1983) had already demonstrated that flanker interference was reduced when the colour of the distractors was made different from that of the target stimulus.

### The spotlight of visual attention

At around the same time that the Eriksen’s introduced their flanker interference task, Charles Eriksen and his colleagues were also amongst the first to start talking about the spotlight of spatial attention (Erikson & Hoffman, 1973; see also Broadbent, 1982; Klein & Hansen, 1990; LaBerge, Carlson, Williams, & Bunney, 1997; Posner, 1980; Posner, Snyder, & Davidson, 1980; Treisman & Gelade, 1980; Tsal, 1983). As Driver and Baylis (1991, p. 102) put it: *“The crux of this metaphor is the idea that space is the medium for visual attention, which selects contiguous regions of the visual field, as if focusing some beam to illuminate an area in greater detail.”*

Now, as might be expected, the notion of a contiguous uniform spotlight of visual attention was soon challenged from a number of directions. On the one hand, researchers, including Charles Eriksen, questioned the limits on its spatial distribution. The spotlight model of attention, and its successors (e.g., Shulman, Remington, & McLean, 1979), was often-discussed in Oxford tutorials. From a fixed spotlight model, with the spotlight also moving at a fixed and, according to Tsal (1983), measurable speed of 8 ms per degree of visual angle (see also Eriksen, & Murphy, 1987; Eriksen & Yeh, 1985; though see Eriksen & Webb, 1989; Kramer, Tham, & Yeh, 1991; Murphy & Eriksen, 1987; Remington & Pierce, 1984; Sagi & Julesz, 1985, for contrary findings) through to an adjustable beam (LaBerge, 1983) or “zoom lens” (Eriksen & St. James, 1986; Eriksen & Yeh, 1985; and other gradient-type models; Shulman, Sheehy, & Wilson, 1986).

The central idea behind Eriksen and St. James’ (1986) “zoom lens” model was that there was a fixed amount of attentional resources that could either be focused intensively over a narrow region of space, or else spread out more widely across the visual field. Subsequently, others came out with the rather more curious-sounding “donut” model (Müller & Hübner, 2002). If you were wondering, the latter was put forward to allow for the finding that attention could seemingly be divided between two different locations simultaneously (e.g., McMains & Somers, 2004; Müller, Malinowski, Gruber, & Hillyard, 2003; Tong, 2004).

Separate from the question of how attention is focused spatially, there was also a question of how attention moved between different locations, as when one probable target location was cued before another. However, as Eriksen and Murphy (1987, p. 303) noted early on when considering the seemingly contradictory evidence concerning whether visual spatial attention moves in a time-consuming and continuous manner or not: *“How attention shifts from one locus to another in the visual field is still an open question. Not only is the experimental evidence contradictory, but the experiments are based on a string of tenuous assumptions that render interpretations of the data quite problematic.”* As we will see below, though, this precautionary warning did not stop others from trying to extend the spotlight-type account beyond the visual modality.

One of the other uncertainties about the spotlight of attention subsequently concerned whether or not Posner’s “beam” (e.g., Posner, 1978, 1980) was the same as Treisman’s “glue” (e.g., Treisman, 1986). Perhaps there were actually multiple spatial spotlights of attention in mind. In this regard, informative research from Briand and Klein (1987) highlighted some important differences. The latter researchers concluding that only exogenous attentional orienting behaved like Treisman’s glue.

### Flanker interference and perceptual load

**Fig. 1:**
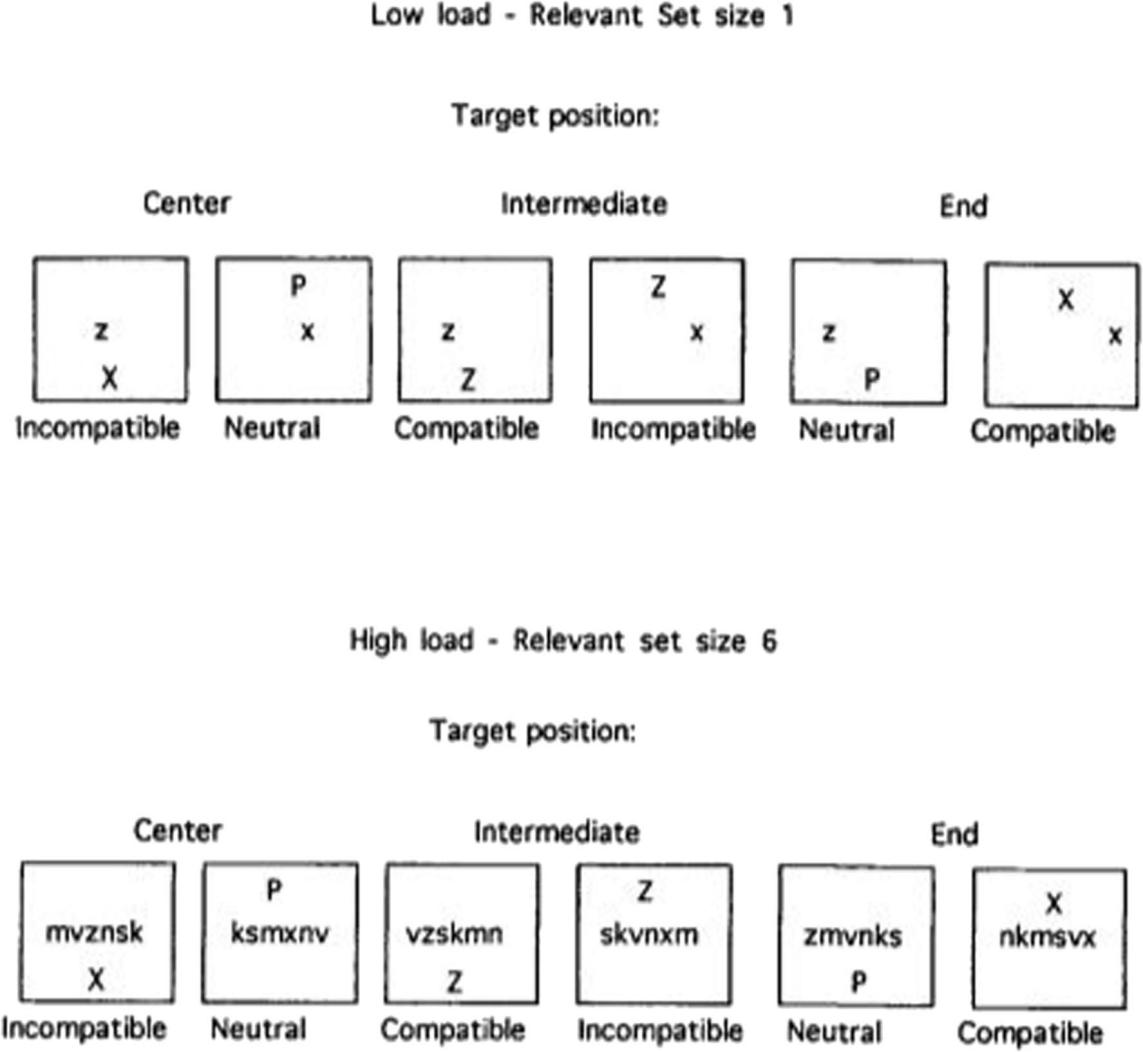
Experimental stimuli used in Lavie’s (1995, Fig. 1) Experiment 1. The participants had to make speeded discrimination responses concerning whether the target letter presented in the middle row of the display was an “X” or a “Z.” Meanwhile a distractor stimulus was presented unpredictably from either above or below the middle row

Lavie (1995; see also Lavie & Tsal, 1994) modelled a number of her early experiments on perceptual load on a modified version of Eriksen flanker task. Here, the perceptual load of the visual task was manipulated by, for example, increasing the number, and/or heterogeneity, of distractors presented in the display (see Fig. 1). The basic idea was that we have a fixed amount of attentional resources that need to be used at any one time (see also Miller, 1991, for an earlier consideration of perceptual load in the context of the Eriksen flanker task). Hence, if processing/perceptual load is high then attentional selection is likely to occur early, whereas if the perceptual load of the primary task is low, late selection might be observed instead. One challenge around load theory relates to the question of how to move beyond a merely operational definition of load. Another challenge has come from those researchers wondering whether attentional narrowing, rather than specifically attentional selection, might explain the results of manipulations of load (e.g., Beck & Lavie, 2005; Van Steenbergen, Band, & Hommel, 2011). The latter concern was often raised in response to the fact that, just as in Eriksen and Eriksen’s (1974) original flanker study, the relevant target stimuli were typically always presented from fixation, or else very close to it.

## Interim summary

Ultimately, the primarily spatial account of attentional selection stressed by much of Eriksen’s early research came to be challenged by other findings that started to emerge highlighting the object-based nature of visual selection (e.g., Baylis & Driver, 1993; Duncan, 1984; Treisman, Kahneman, & Burkell, 1983; Shinn-Cunningham, 2008; Tipper et al., 1991, 1994). While the latter research by no means eliminated the important role played by space in attentional selection, it nevertheless highlighted that in those environments in which objects are present in the scene/display, object-based selection might win out over a straight space-based account (Abrams & Law, 2000; Lavie & Driver, 1996; Richard, Lee, & Vecera, 2008; see Chen, 2012, for a review). Before moving on, it is perhaps also worth noting that while C. W. Eriksen’s interests primarily lay with trying to understand spatial attentional selection in the normal brain, many other researchers, including a number of my former collaborators here in Oxford, subsequently took Eriksen’s approach as a basis for trying to understand how mechanisms of selective attention might suffer following brain damage such as stroke or neglect (see Driver, 1998, for a review).

## Extending Eriksen’s approach beyond vision

Taking the three key ideas from Eriksen’s work that have been discussed so far,^5^ the Eriksen flanker task, the idea of a spatial attentional spotlight (remaining agnostic, for now, about its precise shape), and the notion that it might take time for spatial attention to move from one location to another, I will now take a look at how these ideas were extended to the auditory and tactile modalities in those wanting to study attentional selection beyond vision. One point to highlight at the outset here when comparing the same, or similar, behavioral task when presented in different senses is the differing spatial resolution typically encountered in vision, audition, and touch. For instance, resolution at, or close to, the fovea, where the vast majority of the visual flanker interference research has been conducted to date, tends to be much better than at the fingertip, where the majority of the tactile research has been conducted (see Gallace & Spence, 2014), or in audition. At the same time, however, it is also worth bearing in mind the very dramatic fall-off in spatial resolution that is seen in the visual modality as one moves out from the fovea into the periphery. A similar marked decline has also been documented in the tactile modality when stimuli are presented away from the fingertips (e.g., Stevens & Choo, 1996; Weinstein, 1968), what Finnish architect Juhani Pallasmaa (1996) once called “the eyes of the skin.” One of the most relevant questions, therefore, in what follows, is what determines the resolution of the spatial spotlight in the cases of selection within, and also between, different sensory modalities.

One other related, though presumably not quite synonymous, difference between the spatial senses that is worth keeping in mind here relates to their differing bandwidths. Zimmerman (1989) estimated the channel capacities as 10^7^ bits/s for the visual modality, 10^5^ for auditory modality, and 10^6^ bits/s for touch. However, in terms of effective psychophysical channel capacity (presumably a more appropriate metric when thinking about flanker interference as assessed in psychophysical tasks), Zimmerman estimated these figures at 40 (vision), 30 (audition), and 5 (touch) bits/s (see also Gallace, Ngo, Sulaitis, & Spence, 2012).

### The non-visual flanker task

As researchers started to consider attentional selection outside the visual modality, it was natural to try and adapt Eriksen’s robust spatial tasks to the auditory and tactile modalities – that is, to the other spatial senses (e.g., Chan, Merrifield, & Spence, 2005; Gallace, Soto-Faraco, Dalton, Kreukniet, & Spence, 2008; Soto-Faraco, Ronald, & Spence, 2004). Importantly, however, extending the flanker interference task into the other spatial senses raised its own problems. For instance, as we have just seen, the auditory modality is generally less acute in the spatial domain and more acute in the temporal dimension (e.g., Julesz & Hirsh, 1972; Welch, DuttonHurt, & Warren, 1986). At the same time, however, moving beyond a unimodal visual setting also raises some intriguing possibilities as far as the empirical research questions that could be addressed were concerned (such as, for instance, the nature of the spatial representation on which the spotlight of attention operates).

Chan et al. (2005) adapted the Eriksen flanker task to the auditory modality. These researchers had their participants sit in front of a semi-circular array of five loudspeaker cones. The participant’s task in Chan et al.’s first experiment involved trying to discriminate the identity of the target word (“bat” vs. “bed”) presented from the central loudspeaker situated directly behind fixation, while trying to ignore the identity of the auditory distractor words (spoken by a different person) presented from one of the two loudspeakers positioned equidistant 30° to either side of fixation (note, here, the much larger spatial separation in audition than typically seen in visual studies). The results revealed a robust flanker interference effect, with speeded discrimination responses to the target being significantly slower (and much less accurate) if the distractor voices repeated the non-target (incongruent) word as compared to when repeating the target word instead.^6^

Intriguingly, a second experiment revealed little variation in the magnitude of the auditory flanker interference effect as a function of whether the distractors were placed 30°, 60°, or 90° from the central target loudspeaker (with distance varied unpredictably on a trial-by-trial basis). This result suggests a very different spatial fall-off in distractor interference as compared to what had been reported in Eriksen and Eriksen’s (1974) original visual study. Their response compatibility effects fell off within 1° of visual angle of the target location. One account for such between-modality effects might simply be framed in terms of differences in spatial resolution between the senses involved. However, another important difference between the auditory and the visual versions of the Eriksen flanker interference task that it is important to bear in mind here is that in the former case both energetic and informational masking effects may be compromising auditory performance (Arbogast, Mason, & Kidd, 2002; Brungart, Simpsom, Ericson, & Scott, 2001; Kidd, Jr., Mason, Rohtla, & Deliwala, 1998; Leek, Brown, & Dorman, 1991). By contrast, in the visual studies, any interference is attributable only to informational masking. Note that energetic masking is attributable to the physical overlap of the auditory signals in space/time. Informational masking, by contrast, is attributable to the informational content conveyed by the stimuli themselves (e.g., Lidestam, Holgersson, & Moradi, 2014).

**Fig. 2:**
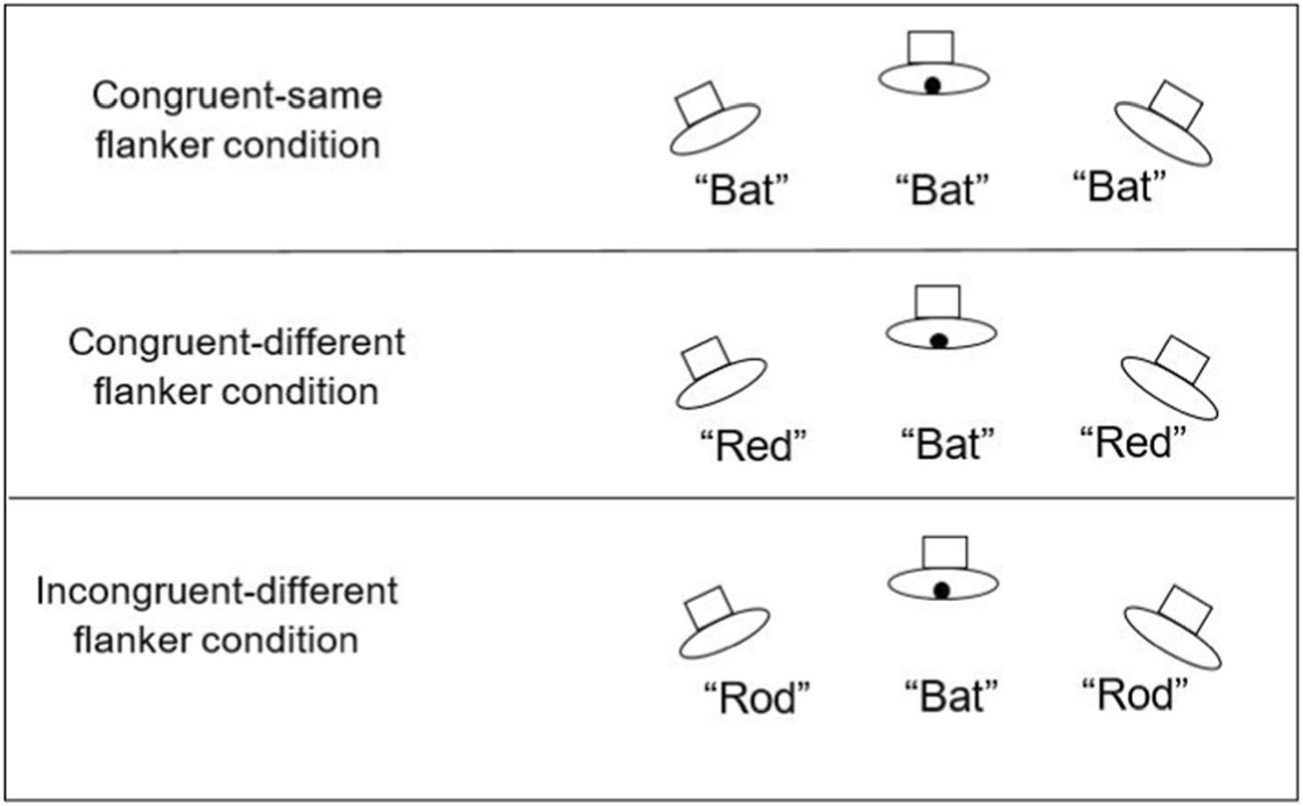
The three experimental conditions used in Chan et al.’s (2005) Experiment 3, an auditory version of the Eriksen flanker task (response key 1 = “Bat” and “Red” Response key 2 = “Rod” and “Bed”)

In order to address the concern over an energetic masking account, in a third experiment Chan and his colleagues (2005) had two words associated with one response and another two words with another response (see Fig. 2). Intriguingly, participants’ performance in the Congruent-same and the Congruent-different conditions was indistinguishable and, in both cases, it was much better than the performance seen in the Incongruent-different condition. This despite the fact that any energetic masking effects should have been matched in the latter two conditions.

Although still present, concerns about the impact of visual fixation on auditory selection have been less of a concern than was the case in the visual modality (though see Reisberg, 1978; Reisberg, Scheiber, & Potemken, 1981; Spence, Ranson, & Driver, 2000c). Nevertheless, when the flanker interference paradigm was adapted to the tactile modality, the targets and distractors have nearly always been presented equidistant from central visual fixation. In this case, the target stimulus was presented to the finger or thumb of one hand while the distractor stimulus was presented to the finger or thumb of the other hand. Once again, robust distractor interference effects were observed (e.g., Soto-Faraco et al., 2004). In the tactile interference case, however, one of the intriguing new questions that we were able to address concerned what happens when the separation between the participant’s hands was varied, while keeping the skin sites stimulated constant. The results of a series of such laboratory experiments demonstrated that it was the separation in external space, rather than the somatotopic separation (i.e., the distance across the skin surface), that primarily determined how difficult participants found it to ignore the vibrotactile distractors. Intriguingly, however, subsequent research using variants of the same intramodal tactile paradigm (Gallace et al., 2008) went on to reveal that compatibility effects could be minimized simply by having the participants respond vocally/verbally rather than by depressing, or releasing, one of two response buttons/foot-pedals (cf. Eriksen and Hoffman, 1972a, b). Notice how the use of a vocal response removes any spatial component from the pattern of responding.

### Does the spotlight of attention operate outside the visual modality?

In recent decades, a number of researchers have taken the spatial spotlight metaphor and extended it beyond the visual modality (e.g., Lakatos & Shepard, 1997; Rhodes, 1987; Rosli, Jones, Tan, Proctor, & Gray, 2009; see also Rosenbaum, Hindorff, & Barnes, 1986). For instance, in the study reported by Rhodes, participants had to specify the location of a target sound by means of a learned verbal label. A series of evenly-spaced locations around the participant were each associated with numbers in a conventional sequence (1, 2, 3, etc.). The latency of the verbal localizing response on a given trial increased linearly with the distance of the target from its position on the preceding trial. Rhodes argued that this increase reflected the time taken to shift the spatial spotlight of attention between locations and therefore implied that attention moved through empty space at a constant rate (as had been suggested previously by Tsal, 1983, for vision; see also Shepherd & Müller, 1989). However, the movement could as well have been along some numerical, rather than spatial, representation.

At this point, it is worth stressing that space is not intrinsically relevant to the auditory modality in quite the same way (Rhodes, 1987). Hence, according to certain researchers, attentional selection is perhaps better thought of as frequency-based rather than as intrinsically space-based (e.g., Handel, 1988a,b; Kubovy, 1988). Yet, at the same time, it is also clear that we do integrate auditory, visual, and tactile stimuli spatially. The vibration I feel, the ringtone I hear, all seem to come from the mobile device I see resting in my palm. That is, multisensory feature binding would appear to give rise to what feels like multisensory object representations. While the phenomenology of multisensory objecthood (O’Callaghan, 2014) is not in doubt (though see Spence & Bayne, 2015), little thought has seemingly been given over to the question of how such binding is achieved, especially in the complex multisensory scenes of everyday life. Think here only of the famous cocktail party situation (see Spence, 2010b; Spence & Frings, 2020). Intriguingly, Cinel, Humphreys, and Poli (2002) conducted one of the few studies to have demonstrated illusory conjunctions between visual and tactile stimuli presented at, or near to, the fingertips.

**Fig. 3:**
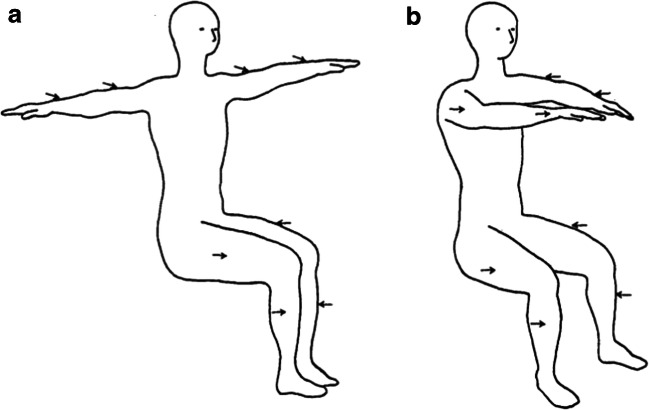
Arrows indicating the position from which air-puff stimuli could be presented in Lakatos and Shepard’s (1997) study of tactile spatial attentional shifts. By varying the participant’s posture, it was possible to demonstrate that it was straight-line distance through space that mattered more to reaction times than necessarily distance across the body surface [Figure reprinted from Lakatos and Shepard (1997, Fig. 3)]

Lakatos and Shepard (1997) asked a similar question in the tactile modality (see Fig. 3). First, one of eight locations was identified verbally. Two seconds later, a second location was also identified verbally. At the same time, air-puff stimuli were presented from four of the eight possible locations distributed across the participant’s body surface. The latter had to respond in a forced choice manner as to whether an air-puff stimulus had been presented from the second-named location. In order to try and ensure that the participants did indeed focus their attention on the first-named location, the first- and second-named locations were the same on 70% of the trials. On the remaining 30% of the trials, the second location was picked at random from one of the remaining seven positions. Once again, the question of interest was whether RTs would increase in line with the distance that the putative attentional spotlight had to move through space. The results revealed a clear linear effect of distance on RTs. In a second experiment, when a different posture was adopted (again see Fig. 3), the results suggested that it was straight-line distance between the named locations that determined RTs.

Given that spatial acuity varies so dramatically across the body surface (e.g., Stevens & Choo, 1996; Weinstein, 1968), one interesting question to consider here is whether similar speeds of movement would also be documented in areas of higher tactile spatial resolution, such as, for example, within the fingers/hand. I am not, however, aware of anyone having addressed the question of what role, if any, spatial resolution has on the speed of the spotlight’s spatial movement across a given representation of space (see also Gallace & Spence, 2014).

Another important issue relates to the differing spatial resolution documented in the different senses. In, or close to, foveal vision, where the vast majority of visual selection studies have been conducted, spatial resolution is undoubtedly much better than for the other spatial senses of hearing and touch. Indeed, the spatial separation between target and distractor locations is always much, much larger in the case of auditory or tactile versions of the flanker task, though typically little mention is made of this fact. The lower spatial resolution when one moves away from the situation mostly studied with foveal vision will likely reduce the signal/noise ratio associated with any given stimulus event, thereby presumably increasing the processing time needed to identify any particular stimulus event. Such issues are clearly important when it comes to a consideration of multisensory selection, as discussed briefly below. To put the question bluntly, one might wonder what is the effective spatial resolution for the spotlight of attention, say, when dealing with multisensory inputs? At the same time, however, it is also worth stressing that in everyday life much of our multisensory information processing presumably takes place outside of foveal vision, where the spatial resolution of the spatial senses (vision, audition, and touch) often turn out to be much more evenly matched.

## Multisensory selection

### The crossmodal congruency task

**Fig. 4:**
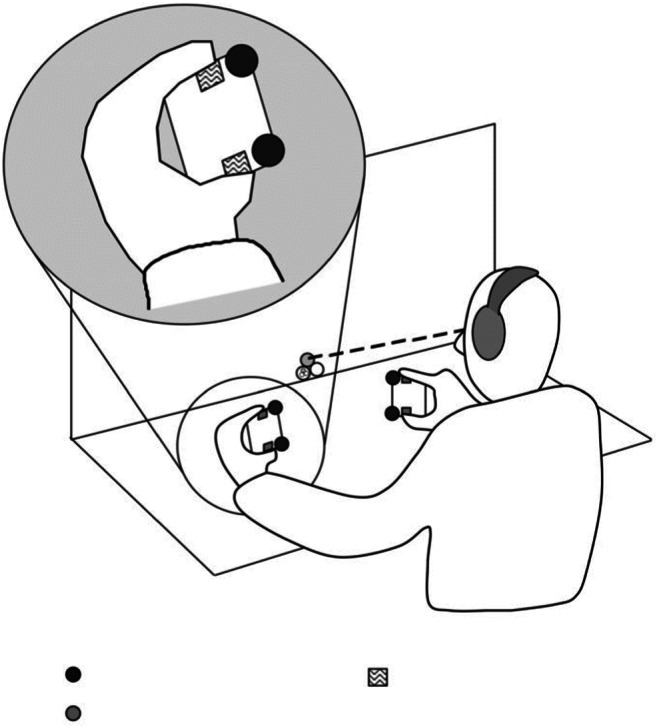
Schematic view of the apparatus and participant in a typical study of the crossmodal congruency task. The participant holds a foam cube in each hand. Two vibrotactile stimulators and two visual distractor lights (zig-zag-shaded rectangles and filled circles, respectively, in the enlarged inset) are embedded in each foam block, positioned next to the participant’s thumb or index finger. Note that white noise is presented continuously over headphones to mask the sound of the operation of the vibrotactile stimulators and foot-pedals. The participants made speeded elevation discrimination responses (by raising the toes or heel of the right foot) in response to vibrotactile targets presented either from the “top” by the index finger of one or the other hand or from the “bottom” by one or the other thumb, respectively

Having taken flanker interference out of the unisensory visual setting into the unisensory auditory and tactile modalities, it then became only natural to ask the question about crossmodal attentional selection in the distractor interference setting. This led to the emergence of the widely used crossmodal congruency task (CCT; Pavani, Spence, & Driver, 2000; Spence, Pavani, & Driver, 1998, 2004b; see Spence, Pavani, Maravita, & Holmes, 2008, for a review). In the basic version of the paradigm, participants are required to discriminate the elevation of vibrotactile targets presented to the index finger or thumb of either hand, while at the same time trying to ignore the visual distractors (so, presented in a different modality from the target) presented from an upper or lower LED situated on either the same or the opposite hand (see Fig. 4). Typically, the onset of the distractors precedes that of the targets by about 30 ms (cf. Gathercole & Broadbent, 1987). A robust crossmodal response compatibility effect, often referred to as the crossmodal congruency effect, or CCE for short, has been documented across a wide range of stimulus conditions.

There have since been many studies using the crossmodal congruency task (first presented at the 1998 meeting of the Psychonomic Society; Spence, Pavani, & Driver, 1998). What is more, similar, if somewhat smaller, interference effects can also be obtained if the target and distractor modalities are reversed such that participants now have to respond to discriminate the elevation of visual targets while attempting to ignore the location of vibrotactile distractors (Spence & Walton, 2005; Walton & Spence, 2004). A few researchers have also demonstrated crossmodal congruency effects between auditory and tactile elevation cues (Merat, Spence, Lloyd, Withington, & McGlone, 1999; see also Occelli, Spence, & Zampini, 2009). Intriguingly, however, one of the important differences between the crossmodal and intramodal versions of the flanker task is that perceptual interactions (i.e., the ventriloquism effect and/or multisensory integration) may account for a part of the distractor interference effect in the crossmodal case (e.g., Marini, Romano, & Maravita, 2017; Shore, Barnes, & Spence, 2006). By contrast, spatial ventriloquism and multisensory integration presumably play no such role in the intramodal visual Eriksen flanker task.

### A multisensory spotlight of attention

Eventually, the spotlight metaphor made it into the world of multisensory and crossmodal attention research (e.g., Buchtel & Butter, 1988; Butter, Buchtel, & Santucci, 1989; Farah, Wong, Monheit, & Morrow, 1989; Posner, 1990; Ward, 1994). In the case of exogenous spatial attention orienting, Farah et al. (1989, p. 462) suggested that there may be *“a single supramodal subsystem that allocates attention to locations in space regardless of the modality of the stimulus being attended, modulating perception as a function of location across modalities”* (see also Spence, Lloyd, McGlone, Nicholls, & Driver, 2000a; Spence, McDonald, & Driver, 2004a; Spence, 2010a). By contrast, in the case of endogenous spatial attention (Jonides, 1981b), much of the spatial attention research subsequently switched the focus to the question of whether the spotlight of attention could be split between different locations, in different modalities simultaneously (e.g., Lloyd, Merat, McGlone, & Spence, 2003; Spence & Driver, 1996; Spence, Pavani, & Driver, 2000b). Much of the experimental evidence supported the view that while endogenous spatial attention could be split between different locations, there were likely to be significant performance costs (picked up as a drop in the speed or accuracy of participants’ responses; see Driver & Spence, 2004, for a review). Such results, note, are seemingly inconsistent with Posner’s (1990) early suggestion that modality-specific attentional spotlights might be organized hierarchically under an overarching multisensory attentional spotlight.

## Conclusions

To conclude, Eriksen’s seminal research in the 1970s and 1980s was focused squarely on questions of visual spatially selective attention in neurologically healthy adult participants. His theoretical accounts of attention operating as a zoom lens undoubtedly generated much subsequent empirical research (e.g., Chen & Cave, 2014). What is more, versions of the Eriksen flanker paradigm have often been used by researchers working across cognitive psychology, and specifically attention research. At the same time, however, a number of esearchers have subsequently attempted to extend Eriksen’s theoretical approach/experimental paradigms out of the visual modality into the other spatial senses, namely audition and touch, but there have been challenges. Researchers, including your current author, have been able to make what seem like useful predictions into the multisensory situations of selection that are perhaps more representative of what happens in everyday life.

Ultimately, therefore, I would like to argue that C. W. Eriksen’s primarily visual focus can, and has, by now been successfully extended to the case of non-visual and multisensory selective attention. At the same time, however, it is important to be cognizant of differences in the representation of space outside vision, as well as the other salient differences in information processing capacity that likely make any simple comparison across the senses less than straightforward.

For the vision scientist, one might want to know what additional insights are to be gained from the extension of the Eriksen flanker task outside its original unisensory visual setting? One conclusion must undoubtedly be that the spotlight of attention should not be considered as operating on the space provided by a given modality of sensory receptors (such as the retinal array). Rather, the spotlight of attention would appear to operate on a higher-level representation of environmental space that presumably results from the integration of inputs from the different spatial senses, presumably incorporating proprioceptive inputs too (see Spence & Driver, 2004). At the same time, however, that also leaves open the question of the spatial resolution of this multisensory representation, given the very apparent differences in resolution that have been highlighted by the various unisensory studies of Eriksen flanker interference in the visual, auditory, and tactile modalities. This remains an intriguing question for future research (see Chong & Mattingley, 2000; Spence & Driver, 2004; Spence, McDonald, & Driver, 2004a, for a discussion of this issue in the context of crossmodal exogenous spatial cuing of attention; cf. Stewart & Amitay, 2015).

### Open Practices Statement

As this is a review paper, there are no original data or materials to share.

## Footnotes

1 Julesz and Hirsh (1972) are amongst the researchers interested in considering the similarities and differences between auditory and visual information processing. See Hsiao (1998) for a similar comparison of visual and tactile information processing.

2 The switch from unisensory to multisensory attention research motivated by my then supervisor, Jon Driver’s, broken TV. The sound would emerge from the hi-fi loudspeakers in his cramped Oxford bedsit giving rise to an intriguing ventriloquism illusion (see Driver & Spence, 1994; Spence, 2013, 2014b). That said, there was also some more general interest emerging at the time in trying to extend the Stroop (Cowan, 1989a, b; Cowan & Barron, 1987; Miles & Jones, 1989; Miles, Madden, & Jones, 1989) and negative priming paradigms (Driver & Baylis, 1993) from their original unisensory visual setting to a crossmodal, specifically audiovisual, one.

3 This distinction is important in terms of Treisman’s FIT, while makes a meaningful distinction, note, between the processing of features and feature conjunctions (e.g., Treisman & Gelade, 1980).

4 The reason, at least according to Driver and Baylis (1991), being that FIT predicts a distance effect for feature conjunctions but not for feature singletons, which are thought to be processed in parallel across the entire display. In fact, under their specific presentation conditions, Driver and Baylis obtained the opposite result, hence seemingly incongruent with Treisman’s account (see also Shulman, 1990).

5 There were, or course, many more findings, but covering any of them more fully falls beyond the scope of the present article.

6 Note that in this study RT and proportion correct were combined into a single measure of performance, namely inverse efficiency (see Townsend & Ashby, 1978, 1983).
